# High Expression of Derlin-1 Is Associated with the Malignancy of Bladder Cancer in a Chinese Han Population

**DOI:** 10.1371/journal.pone.0168351

**Published:** 2016-12-15

**Authors:** Ziyu Wu, Chao Wang, Zhan Zhang, Wenlou Liu, Hengsen Xu, Huanqiang Wang, Yun Wang, Wei Zhang, Shou-Lin Wang

**Affiliations:** 1 School of Public Health, Nanjing Medical University, 101 Longmian Avenue, Nanjing, People's Republic of China; 2 Department of Urology, Huai’an Hospital Affiliated with Xuzhou Medical University, Huai’an, People's Republic of China; 3 Department of Oncology, Zhongnan Hospital of Wuhan University, Wuhan, People's Republic of China; 4 Department of Urology, the First Affiliated Hospital of Nanjing Medical University, Nanjing, People's Republic of China; University of Nebraska Medical Center, UNITED STATES

## Abstract

Derlin-1 is overexpressed in various types of solid tumors and has an important function in cancer progression. However, its expression pattern in and association with the clinicopathological characteristics of human bladder cancer remain unclear. In the present study, 3 pairs of fresh samples of bladder cancer tissue and paracancerous tissue were first detected by liquid chromatography tandem mass spectrometry (UPLC-MS/MS) to screen for differentially expressed proteins. Following bioinformatics analysis and assessments by qRT-PCR and western blotting, Derlin-1 was selected as a candidate protein and was then validated in samples from patients with bladder cancer by immunohistochemistry and western blotting. The results showed that the bladder cancer tissues exhibited higher levels of Derlin-1 expression than the paracancerous tissues (P < 0.05). Positive expression of Derlin-1 was significantly correlated with tumor stage, histological grade, and lymph node metastasis (P < 0.001) but was not correlated with other clinicopathological parameters including patient age (P = 0.758) and gender (P = 0.831). Besides, Derlin-1 was highly expressed in BC cell lines (um-uc-3 and T24), and the interference of Derlin-1 could reverse EMT progression, inhibit the tumor migration and invasion in T24 cells. Further, patients with positive Derlin-1 expression had shorter overall survival than those with negative expression (P < 0.001). Taken together, our results demonstrated that Derlin-1 was overexpressed in bladder cancer and was associated with the malignancy of bladder cancer.

## Introduction

Bladder cancer (BC) is the second most common cancer of the genitourinary system and the second leading cause of mortality from cancer of the genitourinary system [1]. Approximately 386,000 patients worldwide are diagnosed with BC, and approximately 150000 patients die from this disease each year [2]. It is estimated to be the ninth most common malignancy [3]. The majority of bladder tumors arise in the urothelium. As these tumor grow, they secrete angiogenic factors promoting vascular growth to facilitate optimal oxygen and nutrient delivery. However, oxygen and nutrients are not sufficient for tumor growth; thus, these tumors experience worsening nutrient starvation [4, 5]. Endoplasmic reticulum (ER) stress is induced by hypoxia and nutrient deprivation, which may eventually result in cell death by activating multiple apoptotic pathways [6]. Several genetic alterations are involved in tumor development and progression, allowing tumor cells to escape from growth control and apoptosis [7]. It has been reported that cytotoxic insults occur in many cancer cells and that cancer cells often enhance their responses to resist persistent stress [8, 9]. To better cope with stressful microenvironments, cells may evoke cytoprotective responses, such as the ER overload response, to enable them to adapt the unfavorable conditions.

Derlin-1 is a part of the p97 ATPase complex, which mediates the retro-translocation of proteins from the ER lumen into the cytosol and participates in the dislocation of misfolded proteins from the ER [10–12]. Derlin-1 is a multifunctional protein. Accumulating evidence has strongly demonstrated that Derlin-1 functions in cancer progression. Recently, some studies documented that the expression of Derlin-1 increased in six types of human cancers. Antibodies targeting Derlin-1 suppressed colon tumor growth in isogenic mice [13]. In human breast, lung, and colon cancers, elevated expression of Derlin-1 was observed, and was found tobe related to tumor grade and lymph node metastasis [13–15]. Overexpression of Derlin-1 induced cell apoptosis by attenuating ER stress in breast cancer [14]. These findings showed that Derlin-1 may be a new oncogene. Epithelial-mesenchymal transition (EMT) accompanying loss of E-cadherin is important for invasiveness and metastasis of BC [16], current studies showed that EMT played a key role in the initiation and development of metastasis during tumor progression of BC [17]. However, little is known about the role of Derlin-1 in progression of bladder cancer.

In the present study, we explored the expression patterns of Derlin-1 in bladder cancer tissues and further investigated the correlation between bladder cancer and Derlin-1 protein expression as well as the correlation between Derlin-1 expression and clinicopathologic characteristics and prognosis, to better understand its role in tumor biology and its potential implications for cancer progression.

## Materials and Methods

### Patients and samples

Cancerous tissue samples and partly matched paracancerous tissue samples were collected between January 2008 and August 2010 from 144 patients who underwent primary cystectomy or transurethral bladder tumor resection at the Department of Urology, the First Affiliated Hospital of Nanjing Medical University (Nanjing, China). TNM staging and histological grade were classified according to the World Health Organization (WHO) 2004 criteria [18] and the Union for International Cancer Control (www.uicc.org/). None of the patients received radiotherapy or chemotherapy before surgical resection. Follow-up information was obtained by reviewing patient medical records. For all patients, the median follow-up period was 36 months (range, 6–84 months). All tissue specimens were immediately frozen in liquid nitrogen overnight and then stored at -80°C until use. The study was approved by the Institutional Review Board of Nanjing Medical University (IRB00001934), and all the participants signed an informed consent before participating in the study.

### Cell lines and cell culture

The human non-malignant cell line SV-HUC-1, human bladder cancer lines T24 and UM-UC-3 were purchased from Shanghai Institute of Cell Biology, Chinese Academy of Sciences (Shanghai, China), and were cultured in RPMI 1640 medium supplemented with 10% fetal bovine serum under an humidified air atmosphere of 5% CO_2_ at 37°C.

### Analysis of tissue proteomic profiles by Q Exactive UPLC-MS/MS

Three high-grade invasive BC and paracancerous tissues were initially selected for proteomic profile analysis by CapitalBio Corporation (Beijing, China) using ultra-performance liquid chromatography coupled with Q Exactive hybrid quadrupole-orbitrap mass spectrometry (Q Exactive UPLC-MS/MS) (Thermo Fisher Scientific, San Jose, USA), according to previous studies [19–21]. Briefly, tissues were lysed and combined with SDS lysis buffer (4% w/v SDS, 100 mM Tris-HCl, pH 7.6) and then incubated at 95°C for 5 min, briefly sonicated, and centrifuged at 14,000 g for 15 min. The protein concentration of the lysate was determined by BCA assay (Thermo Fisher Scientific). Finally, the lysate was labeled with TMT® Mass Tagging Kits (Thermo Fisher Scientific) and analyzed via Q Exactive UPLC-MS/MS for detection. Data were analyzed by Proteome Discoverer software (Thermo Fisher Scientific).

### Tissue microarray and immunohistochemistry

Bladder cancer tissue microarrays (TMAs) were constructed by a contract service at the Outdo Biotechnology Company Ltd (Shanghai, China). The TMAs contained 31 paraffin-embedded BC and paracancerous tissue samples, as well as 113 pure cancer tissue specimens (175 cores, each core 1.5 mm) from archival patient specimens; complete clinicopathological information regarding these patients was available. Immunohistochemistry was performed using a Bond Polymer Refine Detection kit with a Bond-III automated immunostaining system (Leica Microsystems, Milton Keynes, UK) following the manufacturer’s instructions. In brief, tissue slides were first deparaffinized in xylene, ethanol, and water, and then endogenous peroxidase activity was blocked with 3% hydrogen peroxide in methanol for 10 min. Antigen retrieval was performed in sodium citrate buffer for 2 h. The slides were incubated with rabbit anti-human polyclonal antibodies against Derlin-1 (1:100 dilution, Atlas Antibodies, Sigma-Aldrich, UK) for 2 h at 37°C and then incubated with a biotinylated secondary antibody (DAKO, Denmark) labeled with horseradish peroxidase. PBS, rather than the primary antibody, was used as a negative control. The TMAs were treated with a 0.2% diaminobenzidine (DAB, DAKO, Denmark) solution for 2 min, followed by hematoxylin counterstaining.

The immunostaining results were interpreted independently by two expert pathologists. Staining of Derlin-1 protein in the sections was semiquantitatively scored according to staining intensity and extent. Cytoplasmic immunostaining in tumor cells was considered positive. Intensity was scored as “0” (negative), “1” (weak), “2” (moderate), or “3” (strong). Staining extent was scored as “0” (none), “1” (1–25%), “2” (26–50%), “3” (51–75%), or “4” (76–100%). Staining intensity time was scored from 0–12, and the data regarding Derlin-1 staining were indicative of negative (<4) or positive (>4) expression [22].

### Western blot analysis

The total protein of cancerous and matched paracancerous frozen tissue samples from 10 invasive bladder tumors was extracted in lysis buffer (Vazyme Biotech, Nanjing, China) containing a protease inhibitor (Sigma-Aldrich). Protein concentrations were determined using a Bio-Rad Protein Assay (Bio-Rad, California, USA). Aliquots of total protein (50 μg per lane) were electrophoresed on a 12% SDS-polyacrylamide gradient gel and transferred to nitrocellulose membranes. After being washed in rinse buffer at room temperature and incubated in blocking buffer (5% fat-free milk in rinse buffer) for 30 min, the membranes were incubated for 2 h at room temperature with Derlin-1(1:1000) (Atlas Antibodies, Sigma-Aldrich, UK). After additional washing with rinse buffer, the membranes were incubated with an HRP-conjugated secondary antibody (Santa Cruz) diluted 1:1000 for 2 h at room temperature and subsequently developed with enhanced chemiluminescence reagents (Amershame, Little Chalfont Buckinghamshire, UK). β-actin was used as a reference protein. Qptical densities were analyzed using an ImageMasterTM2D Platinum (Version 5.0, Amersham Biosciences, Piscataway, NJ).

### Cell migration and invasion assays

Once the cells were seeded at 70% confluency, they were then transfected with Derlin-1 siRNA or control (RIBOBIO, Guangzhou, China) for 48 h using Lipofectamine 2000 (Invitrogen, Karlsruhe, Germany).

Cell migration and invasion were assayed using a transwell chamber (Millipore, USA) with and without Matrigel (BD, Franklin Lakes, USA). For the invasion assay, a 8-μm pore size transwell chamber was placed into a 24-well plate coated with 50 μl of 1 mg/ml Matrigel (BD Biosciences) and was incubated for 40 min at 37°C. In both transwell assay, cells suspensions (1 × 10^5^ cells/well) were added to the upper chambers and cultured in medium with RPMI 1640 medium with 2% serum, while 500 μl RPMI 1640 medium containing 10% FBS was added to the lower chambers. The invasion lasted for 24 h at 37°C in a CO_2_ incubator. Cells migrated through the filters were fixed with 100% methanol for 30 min and stained with 0.1% crystal violet for 20 min at room temperature and finally examined and photographed by phase-contrast microscope (Olympus, Tokyo, Japan). The procedure of migration assay was same as the invasion assay described above but filters without coating Matrigel, and the assay lasted for 12 h only.

### Confocal microscopy analysis

T24 cells were transfected with Derlin-1 siRNA or control (RIBOBIO, Guangzhou, China) for 48 h, and then were incubated for fixation and subjected to immunofluorescent analysis by incubation overnight at 4°C with antibodies against Derlin-1(1:100) (Atlas Antibodies, Sigma-Aldrich, UK), E-cadherin or vimentin (1:100, Santa Cruz, CA, USA). After washing twice with PBS, cells were incubated with FITC-conjugated secondary antibodies (1:200, Invitrogen, USA) for 1 h at room temperature, then the cells were stained with DAPI and imaged using laser scanning confocal microscope (LSM710, Carl Zeiss, Thornwood, NY).

### Statistical analysis

All statistical analyses were performed using SPSS Statistics 19.0 software (SPSS Institute, Chicago, IL, USA). For comparisons between 2 groups, statistical significance was determined by Student’s t tests. Associations between Derlin-1 expression and clinicopathological characteristics were analyzed using Pearson chi-square test or Fisher’s exact test. Five-year overall survival (OS) was the primary outcome measure was determined by the Kaplan-Meier method and was analyzed by the log-rank test. A two-tailed P < 0.05 was considered statistically significant.

## Results

### Overexpression of Derlin-1 in BC was identified via UPLC-MS/MS

In the initial study, 3575 proteins were identified in three paired BC samples and in matching paracancerous tissues by Q Exactive UPLC-MS/MS. Proteins exhibiting a minimum 1.5-fold up-regulation or down-regulation in expression between the cancerous and paracancerous samples were given further consideration as possible candidate proteins for further study of bladder cancer progression and prognosis. Ultimately, the screening results showed that 165 proteins were 1.5-fold differentially expressed (P < 0.05), of which 146 proteins were down-regulated, and 19 proteins were up-regulated, with a permutation-based FDR<0.05 (S1 Table). Among the differentially expressed proteins, the expression of Derlin-1 was 1.678-fold higher in BC tissue than in paracancerous tissues (P = 0.020) (S1 Table), which was validated by quantitative real-time PCR (qRT-PCR) and western blotting. Based on bioinformatics analysis and the above results, Derlin-1 was selected as a candidate protein to study the relationships between its expression and the progression and prognosis of bladder cancer.

### Derlin-1 was highly overexpressed in bladder cancer

**Fig 1:**
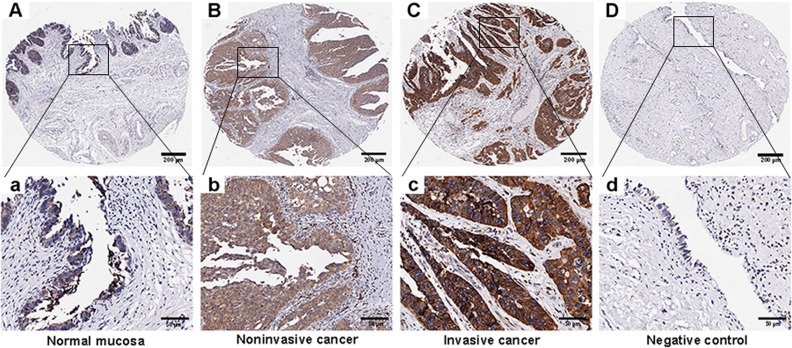
Representative images of Derlin-1 protein expression in IHC microarrays. (A and a), normal mucosa; (B and b), noninvasive cancer; (C and c), invasive cancer; (D and d), negative control; IHC = immunohistochemistry (A, B, C and D, magnification ×40, scale bars: 200 μm; a, b, c and d, magnification ×200, scale bars: 50 μm).

**Fig 2:**
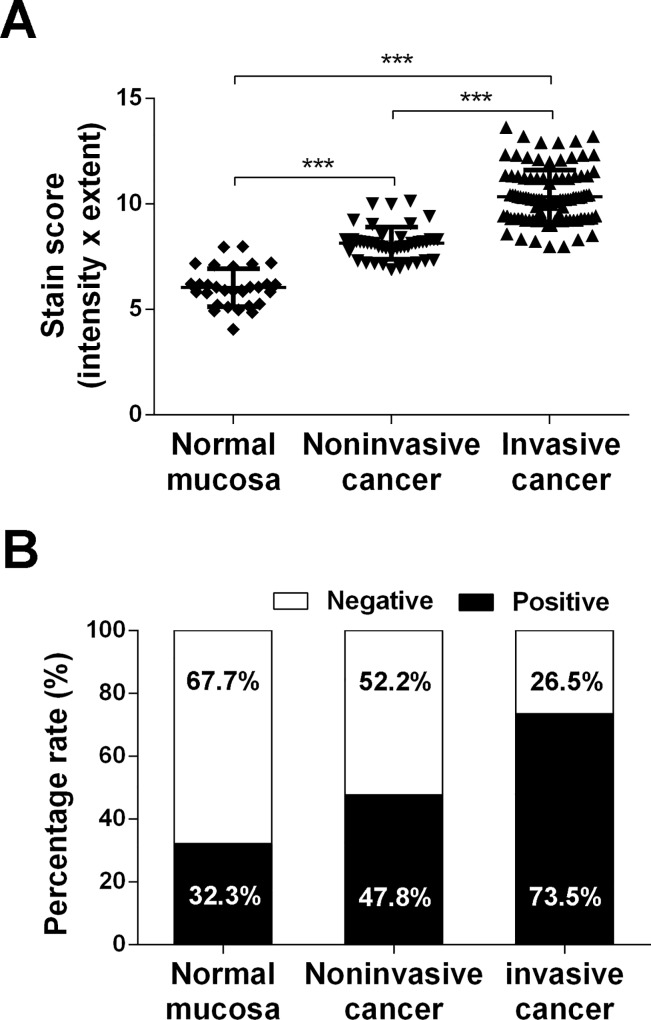
Derlin-1 was highly expressed in bladder cancer tissue microarrays. (A) Scatter plot of Derlin-1 protein staining score in the sections which was semiquantitatively scored according to staining intensity and extent. Staining intensity time received a final score of 0–12. (B) The percentage rate of positive Derlin-1 expression in bladder cancer tissues. Data are expressed as the mean±S.D., ***P < 0.001.

The specificity of the primary antibody against human Derlin-1 was validated by IHC staining in TMAs of bladder cancer tissues and paracancerous tissues. The immunohistochemical staining indicated that Derlin-1 was localized in the cytoplasm in all tissues and was overexpressed in cancer tissues, especially in invasive cancer (Fig 1). In normal bladder mucosa, there was weak cytoplasmic immunostaining. According to the semiquantitative statistical methods mentioned above, the average scores of Derlin-1 protein expression in normal bladder mucosa, noninvasive cancer, and invasive cancer were 6.04, 8.15, and 10.34, respectively (Fig 2A). Derlin-1 protein expression was significantly up-regulated in cancer tissues compared with normal mucosa (P<0.001), especially in invasive bladder cancer (Fig 2A). Moreover, positive Derlin-1 expression was more frequent in invasive bladder cancer (72 of 98 cases; 73.5%) than in noninvasive bladder cancer (22 of 46 cases; 47.8%) or normal bladder mucosa (10 of 31 cases; 32.3%) (Fig 2B).

**Fig 3:**
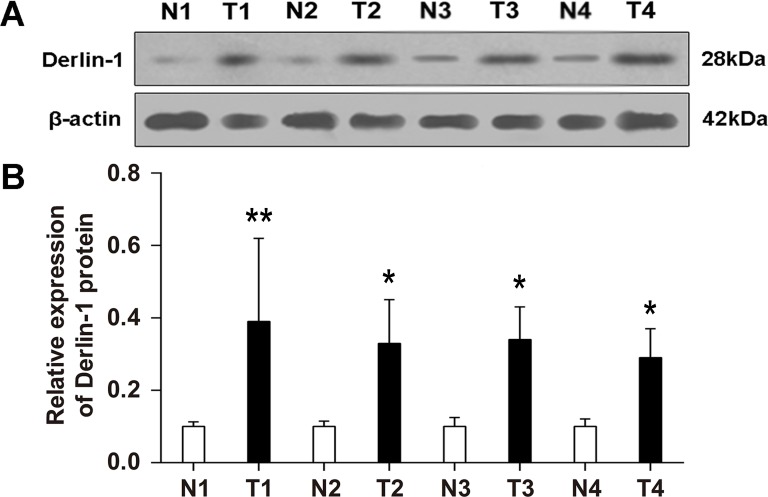
Derlin-1 protein expression in normal mucosa and bladder cancer. (A) Representative graphic of western blot analysis of Derlin-1 protein expression in bladder cancer tissues (T) and paracancerous tissues (N) from four patients. (B) Statistical result. *P < 0.05, **P < 0.01.

**Fig 4:**
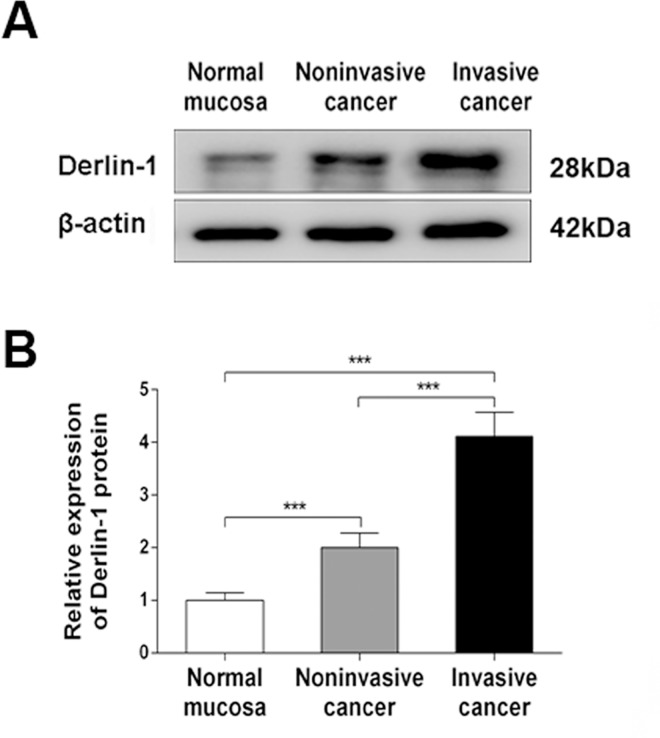
Expression of Derlin-1 protein in noninvasive and invasive bladder cancer tissues. (A) Derlin-1 expression by western blotting. (B) Quantitative analysis of Derlin-1 with respect to β-actin level. Data are expressed as the mean ± S.D., *P < 0.05, **P < 0.01, ***P < 0.001.

Furthermore, the western blotting results showed that Derlin-1 expression in bladder cancer tissue was significantly higher than that in paracancerous tissues at the protein level (all P < 0.05) (Fig 3), which is consistent with the immunohistochemical data. In addition, Derlin-1 was much more highly expressed in invasive cancer than that in noninvasive cancer tissues (Fig 4), indicating that Derlin-1 was potentially involved in the progression of bladder cancer.

### Derlin-1 was associated with clinicopathological characteristics

The correlations between Derlin-1 expression and a series of clinicopathological characteristics, including age, gender, tumor stage, histological grade, and lymph node metastasis, were determined. As shown in Table 1, increased expression of Derlin-1 was significantly correlated with tumor stage (P < 0.005), histological grade (P < 0.001), and lymph node metastasis (P < 0.001), but not with the patient age (P = 0.758) and gender (P = 0.831).

**Table 1:** Derlin-1 immunohistochemical scores in relation to clinicopathological characteristics

Variables	Case (n)	Derlin-1 expression		*P* Value
		Negative (%)	Positive (%)	
Age (years)^a^				0.758
<70	81	29(35.8)	52(64.2)	
≥70	63	21(33.3)	42(66.7)	
Gender				0.831
Female	39	13(33.3)	26(66.7)	
Male	105	37(35.2)	68(64.8)	
Tumor stage^b^				<0.005
pTa~1	46	24(52.2)	22(47.8)	
pT2~4	98	26(26.5)	72(73.5)	
Histological grade				<0.001
Low	51	46(90.2)	5(9.8)	
High	93	4(4.3)	89(95.7)	
lymph node metastasis				<0.001
Negative	89	45(50.6)	44(49.4)	
Positive	55	5(9.1)	50(90.9)	

^a^ Median, 67 years; range, 33–92 years

^b^ Staging and grading according to the WHO 2004 classification system.

### Derlin-1 promoted the malignancy, migration and invasion of bladder cancer in T24 cells

**Fig 5:**
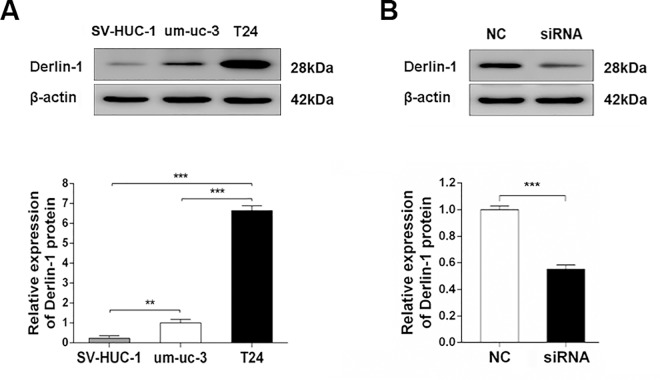
Expression of Derlin-1 protein in human bladder cell lines. (A) Expression of Derlin- protein using western blotting in SV-HUC-1 cells, um-uc-3 cells and T24 cells. (B) Interfere of Derlin-1 in T24 cells. Cells were transfected with nontargeting siRNA or Derlin-1 siRNA for 48h, and the protein expression was detected by western blotting. NC, siRNA negative control; siRNA, Derlin-1 siRNA. Data are expressed as the mean ± S.D., **P < 0.01, ***P < 0.001.

**Fig 6:**
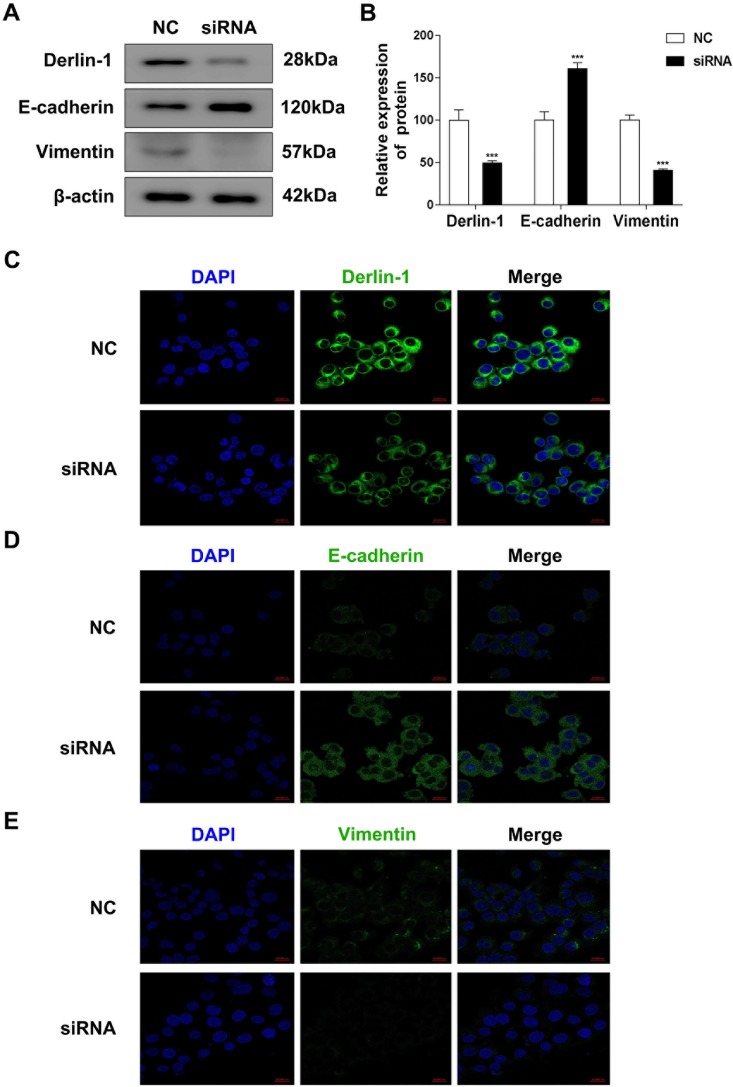
Effects of Derlin-1 on epithelial–mesenchymal transition (EMT) in T24 cells. (A-B) Expressions of E-Cadherin and Vimentin by Western blot assay in T24 cells transfected with Derlin-1 siRNA. β-actin was probed as the loading control. ***P<0.001, compared with negative control. (C-D) Expression of Derlin-1, E-Cadherin and Vimentin (*red*) by immunofluorescence staining analysis using confocal in T24 cells transfected with Derlin-1 siRNA. The nuclei were stained with DAPI (*blue*). scale bar: 5 μm.

**Fig 7:**
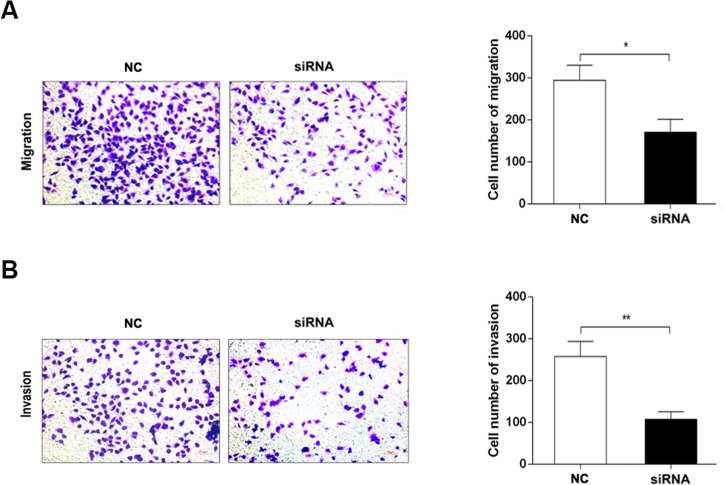
Effects of Derlin-1 on the migration and invasion in T24 cells. (A) Effects of Derlin-1 on cellular migration ability. (B) Effects of Derlin-1 on cellular invasion ability. Representative microscopy images of the migration and invasion assay are shown as ×100. NC, siRNA negative control; siRNA, Derlin-1 siRNA. *P < 0.05, **P < 0.01.

Since most of bladder cancers were transitional cell carcinoma, two kinds of transitional cell carcinoma cell lines, um-uc-3 and T24, were used in the functional study. Derlin-1 was highly expressed in um-uc-3 cells and T24 cell compared with immortalized human urinary tract epithelial cells (SV-HUC-1), especially in T24 cells (6.646-fold higher than in um-uc-3 cell) (Fig 5A). As shown in Fig 5B, Derlin-1 siRNA decreased the expression of Derlin-1 by a half in T24 cells in comparison of siRNA-control. Knockdown of Derlin-1 inhibited the expression of vimentin whereas increased E-cadherin expression by Immunoblotting assay (Fig 6A and 6B) and immunofluorescent analysis (Fig 6C, 6D and 6E), resulting in a correlation between Derlin-1 and EMT. In addition, transwell assays showed that T24 cells transfected with siRNA-Derlin-1 showed a much more weak migration and invasion ability than the cells transfected with siRNA-control (Fig 7).

### Derlin-1 shortened overall survival in patients with bladder cancer

**Fig 8:**
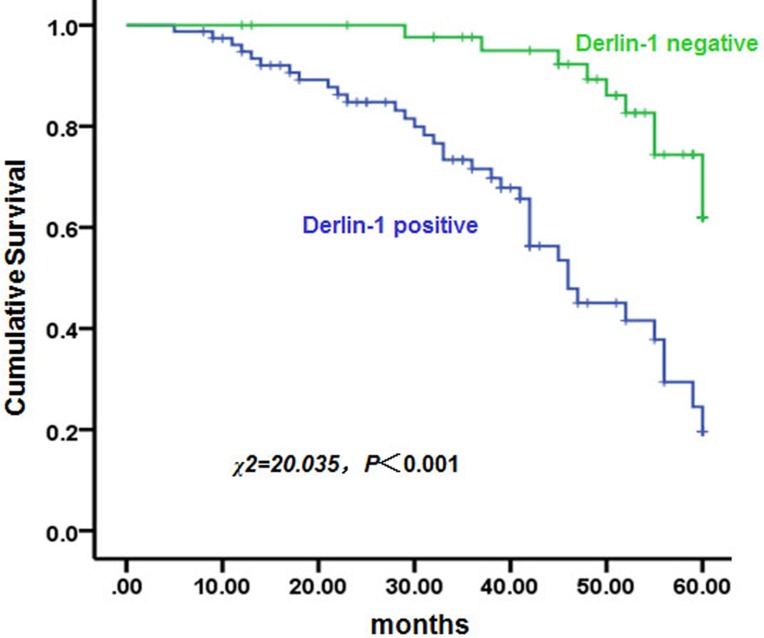
Analysis of 5-year overall survival in patients with positive or negative Derlin-1 expression. Kaplan–Meier survival analysis was used to compare 5-year overall survival between patients with positive or negative Derlin-1 expression. P < 0.001, compared with patients with negative Derlin-1 expression.

Five-year overall survival (OS), as indicated by Kaplan-Meier survival curves of negative and positive Derlin-1 expression, is shown in Fig 8. The median 5-year OS periods of patients with positive or negative Derlin-1 expression were 46 months (95% CI: 36.3 to 55.7 months) and 56 months (95% CI: 51.4 to 60.6 months), respectively. The 5-year OS rate in patients with positive Derlin-1 expression was significantly lower than that in patients with negative Derlin-1 expression (N = 144, P < 0.001) (Fig 8).

## Discussion

Derlin-1 (Derl-1) is a transporter protein that exports of misfolded proteins from the endoplasmic reticulum (ER) and an inhibitor of ER stress-induced apoptosis. Derlin-1 reportedly carries four transmembrane domains, whose N-termini and C-termini are both located within the cytosol. Derlin-1 expression was increased by inducers of ER stress in yeast [23] and *Caenorhabditis elegans* [11]. Almost all solid tumors growth depends on an intact unfolded protein response and tolerance of ER stress, such as hypoxia [24–26]. In human breast, lung, and colon cancer, several lines of direct evidence demonstrated a significant association between Derlin-1 up-regulation and tumor grade, as well as between Derlin-1 up-regulation and lymph node metastasis [14, 15, 27].

Derlin-1 is overexpressed in various types of cancer and to is related to cancer progression [13–15]. However, there has been little research focusing on the expression pattern of Derlin-1 in BC and the role of the protein in BC progression. In the present study, the results of the differential proteomic profiles in BC tissues obtained by Q Exactive UPLC-MS/MS showed that the expression of Derlin-1 was 1.678-fold higher in BC tissues than in paracancerous tissues (P = 0.020). Immunohistochemically, Derlin-1 expression increased gradually from normal tissue to noninvasive cancer to invasive cancer, where its expression was strongest. The percentages of Derlin-1 expression were 32.3% (10 of 31 cases), 47.8% (22/46), and 73.5% (72 of 98 cases) in normal bladder tissues, noninvasive BC tissues and invasive BC tissues, respectively. These data demonstrate that the levels of Derlin-1 protein expression were elevated in the majority of BC tissues compared with normal bladder tissues. Our results are consistent with those of previous studies showing that Derlin-1 is overexpressed in various types of human cancers [13–15, 27].

The level of Derlin-1 expression in higher-grade breast cancers has been demonstrated to be greater than that in lower-grade tumors [14], which suggests that Derlin-1 expression may be associated with a more malignant phenotype. Further studies are needed to elucidate whether the expression of Derlin-1 is indicative of malignant phenotypes of bladder cancer. Our results show that expression of Derlin-1 protein was up-regulated in bladder cancer tissues, which is consistent with the expression pattern demonstrated by immunohistochemical analysis, suggesting that up-regulation of Derlin-1 plays an important role in bladder cancer.

Recent studies have shown that Derlin-1 expression correlates with tumor grade and lymph node metastasis in different types of malignant tumors [14, 15]. In this study, Derlin-1 was much more highly expressed in invasive cancer than that in noninvasive cancer, and overexpression of Derlin-1 significantly correlated with tumor stage, histological grade, and lymph node metastasis, which is consistent with previous reports. Significant associations were observed between Derlin-1 expression and lymph node metastasis, indicateing that Derlin-1 may be associated with aggressive tumor growth or metastasis. A previous study showed that expression of Derlin-1 increases in human breast carcinoma and protects cancer cells from apoptosis induced by ER stress, suggesting that it may confer metastatic properties to cancer cells [14]. Moreover, inducers of ER stress increased the expression of Derlin-1 and the translocation of misfolded proteins from the ER lumen to the cytosol, which is mediated by Derlin-1 [11, 28, 29]. Therefore, based on our study, we speculate that Derlin-1 expression may protect cancer cells from stresses encountered during bladder tumor growth.

The next question to be addressed was whether expression of Derlin-1 was associated with clinical outcomes in bladder cancer. In our study, positive Derlin-1 expression was associated with poor 5-year OS in bladder cancer, which is consistent with the evidence provided by Dong et al. [15], In addition, Derlin-1 was highly expressed in BC cell line T24, and interfere of Derlin-1 led to the decrease in cell migration and invasion in T24 cells, indicating that Derlin-1 may play an essential role in tumor progression and metastasis. A limitation of the present study is that it involved only correlative observations of the relationship between Derlin-1 expression and clinicopathological parameters in bladder cancer and lacked direct evidence regarding the function and underlying mechanism of the effects of Derlin-1. Furthermore, validation of the predictive significance of Derlin-1 requires large-scale studies of homogenous populations.

In conclusion, our results provide evidence that Derlin-1 is overexpressed in bladder cancer and that high expression levels of Derlin-1 correlate with tumor grade, metastasis, and poor overall survival. However, further in-depth studies are necessary to explore the role of Derlin-1 in tumor invasion and metastasis and in other aspects of bladder cancer progression, as well as to elucidate the molecular mechanisms underlying the involvement of Derlin-1 in these processes, which may broaden our knowledge regarding Derlin-1 as an oncogene and as a potential therapeutic target for bladder cancer.

## Supporting Information

S1 Table Differentially expressed proteins in three paired bladder cancer and paracancerous tissues detected by Q Exactive UPLC-MS/MS. A total of 165 proteins were 1.5-fold differentially expressed (P < 0.05), of which 146 proteins were down-regulated, and 19 proteins were up-regulated (FDR < 0.05).
(XLS)
